# Acetylcholine Mediates Dynamic Switching Between Information Coding Schemes in Neuronal Networks

**DOI:** 10.3389/fnsys.2019.00064

**Published:** 2019-11-12

**Authors:** James P. Roach, Bolaji Eniwaye, Victoria Booth, Leonard M. Sander, Michal R. Zochowski

**Affiliations:** ^1^Neuroscience Graduate Program, University of Michigan, Ann Arbor, MI, United States; ^2^Department of Physics, University of Michigan, Ann Arbor, MI, United States; ^3^Department of Mathematics, University of Michigan, Ann Arbor, MI, United States; ^4^Department of Anesthesiology, University of Michigan, Ann Arbor, MI, United States; ^5^Center for the Study of Complex Systems, University of Michigan, Ann Arbor, MI, United States; ^6^Biophysics Program, University of Michigan, Ann Arbor, MI, United States

**Keywords:** acetylcholine, neuronal excitability, information coding, neuromodulation, networks

## Abstract

Rate coding and phase coding are the two major coding modes seen in the brain. For these two modes, network dynamics must either have a wide distribution of frequencies for rate coding, or a narrow one to achieve stability in phase dynamics for phase coding. Acetylcholine (ACh) is a potent regulator of neural excitability. Acting through the muscarinic receptor, ACh reduces the magnitude of the potassium M-current, a hyperpolarizing current that builds up as neurons fire. The M-current contributes to several excitability features of neurons, becoming a major player in facilitating the transition between Type 1 (integrator) and Type 2 (resonator) excitability. In this paper we argue that this transition enables a dynamic switch between rate coding and phase coding as levels of ACh release change. When a network is in a high ACh state variations in synaptic inputs will lead to a wider distribution of firing rates across the network and this distribution will reflect the network structure or pattern of external input to the network. When ACh is low, network frequencies become narrowly distributed and the structure of a network or pattern of external inputs will be represented through phase relationships between firing neurons. This work provides insights into how modulation of neuronal features influences network dynamics and information processing across brain states.

## Introduction

Acetylcholine (ACh) is an important regulator of neural excitability that is essential for brain processes ranging from sleep to cue detection (Marrosu et al., 1995; Parikh and Sarter, 2008). Of its various effects, ACh modulates the excitability of neurons by its interaction with the muscarinic receptor system, which activates a G-protein signaling cascade (Marrion, 1997). An important downstream target of these signals are slow non-inactivating potassium channels. These channels, and their corresponding ionic current (I_M_), are blocked when ACh is high and are responsible for a switch between integrator and resonator excitability types (Prescott et al., 2008).

ACh modulation of I_M_ exerts continuous control of neuronal excitability properties. On the extremes of this range are two predominant excitability types: Type 1 or Type 2. These two excitability types differ in the dynamical mechanism of spike generation. A detailed mathematical analysis can be found (Izhikevich, 2007), but in short Type 2 neurons have increased competition between depolarizing and hyperpolarizing currents which must be overcome to initiate a spike, while Type 1 neurons do not. This leads to several differences in excitability characteristics between the two types, most notably Type 1 neurons initiate firing through a saddle-node on the limit cycle bifurcation while Type 2 neurons initiate firing through a Hopf bifurcation (Gutkin and Ermentrout, 1998; Gutkin et al., 2003).

**Figure 1:**
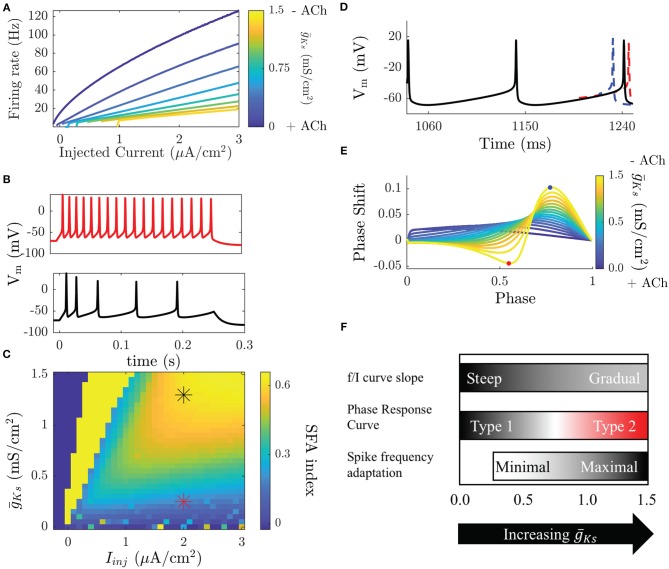
Modulation of neuronal properties in a model of cholinergic modulation. **(A)** The f/I curve increases its slope as ACh increases (${\bar{g}}_{Ks}$ decreases). Blue colors represent the high ACh case. The onset of spike frequency adaptation in the Ks model occurs at a high ${\bar{g}}_{Ks}$. SFA is quantified here by the SFA index, which compares the inter-spike interval between the first two and the last two spikes in an induced burst. (**B**, top) When ${\bar{g}}_{Ks}$ is low SFA is minimal and ISIs are equivalent throughout the burst. (**B**, bottom) When ${\bar{g}}_{Ks}$ is high ISIs gradually increase though out the burst. **(C)** Measured SFA indices for various ${\bar{g}}_{Ks}$ and injected current values show that SFA is only significantly reducing frequency during the burst above ${\bar{g}}_{Ks}$ = 0.25 mS/cm^2^, below this the effects are negligible. Stars indicate the parameters of the voltage traces shown in **(B)**. Dark blue squares indicate parameters that do not elicit spikes and bright yellow squares parameters that yield <3 spikes. **(D)** The PRC is measured by comparing perturbed vs. unperturbed periods when neurons fire at a fixed frequency. When the next spike is earlier the phase response is positive (blue), when it is delayed it is negative (red). **(E)** Type 1 neurons have a strictly positive PRC (blue) while Type 2 neurons have a biphasic PRC. **(F)** Transitions in biophysical properties in the Ks model occur over different ranges of ${\bar{g}}_{Ks}$. Modulation of the f/I slope occurs continuously over the range of ${\bar{g}}_{Ks}$. The slope is steep for low ${\bar{g}}_{Ks}$ and gradual for high ${\bar{g}}_{Ks}$. The transition between a Type 1 and a Type 2 PRC occurs for high ${\bar{g}}_{Ks}$, though the PRC shape does change in a continuous manner as ${\bar{g}}_{Ks}$ changes. SFA has little effect for low ${\bar{g}}_{Ks}$ and only significantly effects the frequency of neurons for high ${\bar{g}}_{Ks}$.

The two characteristics that undergo most dramatic change with the excitability type are the frequency response to an injected constant current and the phase response curve (PRC) (Stiefel et al., 2008a). In terms of spike frequency response to an injected current curve (or a gain function) (Tsuno et al., 2013), both types have a critical current, I_c_, below which no spiking occurs, but are quite different in terms of spiking response around this point. Type 1 neurons will fire at arbitrarily small frequencies as the critical value of I_c_ is reached leading to a continuous curve, whereas Type 2 neurons have a discontinuous frequency increase from quiescence and initiate firing at a higher frequency (Figure 1A). Another critical feature difference between Type 1 and Type 2 neurons is that Type 2 neurons vary their firing rate much less in response to changes in injected current, or have reduced gain (Tsuno et al., 2013). The difference in gain between these neuron types leads to larger differences in firing rates between cells receiving different inputs in Type 1 networks compared to Type 2 networks.

A concurrent change in excitability that occurs with activating the ion channels associated with the M-current is differential response to brief and weak stimuli in terms of spike timing perturbation (i.e., advance or delay). This cellular property is quantified by the PRC (Stiefel et al., 2008a). The PRC is measured, both experimentally and numerically, by driving a neuron to fire at a stable periodic frequency and delivering small, brief, and depolarizing perturbations between its spikes, at different timings (phases) within the spiking cycle. In response to these perturbations the timing of the following spike will be earlier, later, or the same as an unperturbed period (Figures 1D,E). Type 1 and Type 2 neurons display significant differences in PRC shape. A Type 1 PRC is uniformly positive, meaning that perturbations will always advance the timing of the next spike. Type 2 neurons have a biphasic PRC, meaning that depending on the timing of the perturbation it will either advance or delay the next spike. The biphasic character of the Type 2 PRC allows these neurons to synchronize spike firing due to the ability to either shorten or elongate the period, with zero value of phase response becoming a stable fixed point of the dynamics.

In addition to controlling membrane excitability type of a neuron, the changes in I_M_ also regulate spike-frequency adaptation (SFA) (Tang et al., 1997). SFA effectively represents a negative feedback on neuronal firing and is frequently due to a hyperpolarizing current that builds up as a neuron fires action potentials. Here, I_M_ acts as an adaptation current and its blockade causes a significant reduction in SFA (Figures 1B,C). The effects of SFA and gain modulation are related by the fact that neuronal gain shows the firing rate of a neuron when the M-current has saturated. Here, we refer to SFA as the short-time scale effect of reducing the frequency of a neuron as it fires, possibly terminating a burst of firing.

In this article, we argue that this modulation of excitability properties by ACh facilitates a transition in the mode for coding external inputs or network structural features from rate coding when ACh is high to phase coding when ACh is low. Namely, both the PRC and frequency gain describe how neurons change their dynamics of spiking in response to synaptic input. Our previous work extensively studied how these cellular changes affected network wide spatio-temporal pattern formation (Bogaard et al., 2009; Fink et al., 2011, 2013; Roach et al., 2015, 2016; Knudstrup et al., 2016; Mofakham et al., 2016). Now, we are further proposing that these changes in spiking patterns may underlie even more profound changes in the network. We argue that the biophysical features controlled by I_M_ activation are responsible for how network firing patterns interact with external input and characteristics of the physical structure of the network. This, in turn, leads to a dramatic switch in the coding strategy within the same network.

The two predominant coding strategies identified in the brain are rate coding and phase coding. Rate coding represents information in the firing rates between neurons and phase coding represents information in the time differences between neuron firings (Gray and Singer, 1989; Theunissen and Miller, 1995; Von der Malsburg, 1999; Nadasdy, 2010; Ainsworth et al., 2012). While there are examples of rate coding and phase coding existing in the same neural circuits (Jeewajee et al., 2013; Luczak et al., 2015), the ability of networks to switch coding regimes by modulation of the biophysical properties of neurons has yet to be investigated.

We believe that this switch may primarily take place during transition in sleep-wake behavioral phases, as during wake and REM sleep ACh levels remain high, but during NREM sleep they are significantly reduced (Jones, 2005). Recent results suggest that phase coding may play a significant role during network activity and information processing in NREM sleep. For example, studies have shown that abolition of oscillatory activity during NREM sleep, which could be crucial for stabilizing the phase relationships between the neurons, leads to lack of memory consolidation after contextual fear conditioning (CFC) exposure in mice (Ognjanovski et al., 2017). Furthermore, neural frequency changes observed after NREM sleep among heterogeneous populations of neurons (Clawson et al., 2018) could be most naturally explained by spike timing dependent plasticity (STDP) taking place on firing patterns established through phase representation of information during NREM sleep.

Below we demonstrate the possible transition from rate coding to phase coding as ACh changes and provide mechanistic underpinnings for matching representation of information in both regimes.

## Results

In a network, the I_M_ mediated switch between Type 1 to Type 2 excitability together with effects of SFA have a profound influence on resulting network dynamics. Using numerical modeling we've investigated how transitioning neurons from Type 1 to Type 2 excitability impact the patterns of neural activity in networks. Much of our modeling work has used a conductance based neuron model of cholinergic modulation (Stiefel et al., 2008b). The details of this model are included in the Methods section. This biophysical model reproduces the effects of ACh blocking the I_M_ through activation of the M1 acetylcholine receptor. Throughout this paper it will be referred to as the Ks model, named for the slow potassium conductance, ${\bar{g}}_{Ks}$, responsible for the transition from Type 1 to Type 2. Specifically, low ${\bar{g}}_{Ks}$ corresponds to the high ACh, Type 1 excitability condition.

When ACh is high, neurons in the network are Type 1, and the f/I curve increases continuously from 0 Hz with a steep slope as a function of input currents between neurons (Figure 1A). This will result in a wide distribution of firing frequencies across the network when cells are driven by heterogeneous synaptic input or external drive. The resulting frequency distribution will be stable through time, due to reduced SFA during high ACh conditions (low ${\bar{g}}_{Ks}$, Figures 1B,C). On the other hand, during low ACh conditions (high ${\bar{g}}_{Ks}$), when Type 2 excitability dominates, variations in input across the network create less variance in neuronal firing rates due to the shallow slope of the f/I curve. As the firing rates are more uniform, oscillatory firing paired with the increased synchronizability demonstrated by the shape of the PRC (Figures 1D,E) leads to synchronized bursting. The variations in inputs are now reflected by relative phases of firings among interacting neurons, rather than by their frequency variations. Through the changes in neural excitability controlled by the M-current, the circuit is thus shifted between these two, distinct functional regimes: rate coding when ACh is high (low ${\bar{g}}_{Ks}$, Type 1) and phase coding when ACh is low (high ${\bar{g}}_{Ks}$, Type 2; these changes are summarized in Figure 1F).

Much of our previous work on the different dynamics displayed by Type 1 vs. Type 2 networks indicates that Type 1 networks are more sensitive to variations in network structure (Bogaard et al., 2009). Specifically, Type 1 networks have higher variability in neuronal frequency and our results suggest that the particular frequency distribution of these networks will be highly dependent on a particular physical network realization (Roach et al., 2015). Type 2 networks, on the other hand, have more uniform firing rate distributions leading to more synchronous dynamics, suggesting that the effect of the specific network structure will be seen in the phase relationships between neurons. To provide an initial test of this prediction we generated a set of unique networks based on the Watts-Strogatz network model (Watts and Strogatz, 1998). The networks were composed of two interconnected ring lattices, one excitatory and one inhibitory. Since the Watts-Strogatz model of network generation is based on random processes, specific network structures (i.e., sets of inputs and outputs for each neuron) can be reproduced by changing the seed in a random number generator. We generated 20 network realizations; each network structure was simulated 50 times for a given ${\bar{g}}_{Ks}$, randomizing voltage and gating variable initial values each time. This allowed us to compare firing patterns between the 50 runs on the same network realization with the 950 runs on the other network realizations. Additionally, to examine the effects of changing patterns of inputs, a parallel line of simulations were run on a unchanging network structure but with randomized patterns of external applied current (DC) inputs applied across the cells in the network. We generated 20 DC patterns and simulated each 50 times for a given ${\bar{g}}_{Ks}$ value and random initial conditions.

We first investigated how the firing patterns changed when network connectivity structure is varied. In the absence of variations in external input between neurons, patterns in network activity should reflect the specific structure of the network. The aim of these simulations is to show that, for each network topology, for high ACh (low ${\bar{g}}_{Ks}$) a neuron's firing rate will be more correlated on the 50 runs with the same network realization than on the 950 runs with the other network realizations, but that this effect will be reduced as ACh falls. In terms of relative phases of firing between neurons, the opposite will occur. Namely, when ACh is low (high ${\bar{g}}_{Ks}$), the pairwise phase relationships between neurons will be more correlated on the same network realization compared to the other realizations and that this specificity is reduced as ACh increases.

**Figure 2:**
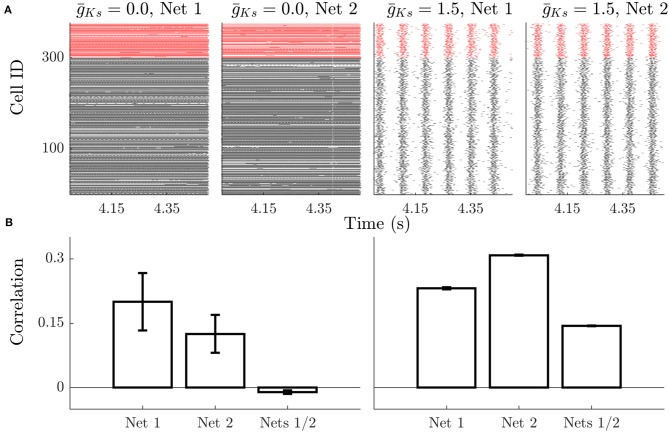
Example dynamics show rate specificity during high ACh dynamics and high phase specificity during low ACh dynamics. **(A)** Raster plots show that high ACh networks have high similarity in firing rates, but low temporal organization. Changes in network structure (Net 1 vs. Net 2) alter which neurons are high frequency vs. low frequency, but this is stable between simulations on the same network. Low ACh networks have a more uniform firing rate but more temporal organization and synchrony. The phase relationships between neurons is stable across stimulations, but not across networks. Black rasters indicate the spike time of excitatory neurons and red rasters indicate inhibitory spikes. **(B)** During High ACh conditions the firing rate of neurons is highly correlated during simulations run on the same networks and uncorrelated between runs on different networks (left). The order of neuron firing during bursts is higher between runs on the same network compared with runs on different networks during low ACh conditions (right). Error bars indicate s.e.m.

This effect is apparent when examining raster plots of network activity. Spiking dynamics for low ${\bar{g}}_{Ks}$ lack temporal organization (Figure 2A) and neurons have variable firing rates (revealed by density of points on the raster plot). The raster plots show that the firing rate pattern is dependent on the network structure for low ${\bar{g}}_{Ks}$, with cells exhibiting different rates in different networks. For high ${\bar{g}}_{Ks}$ firing rates are more uniform as networks enter a bursting regime. Here the frequencies of cells across the network are highly similar, but the organization of neurons within bursts is more consistent across runs on the same network realization and changes for different network realizations. This result is summarized on Figure 2B; for low ${\bar{g}}_{Ks}$ networks the frequency correlation of neurons is high on the same network structure and very low across structures (Figure 2B, left). When comparing burst structure in high ${\bar{g}}_{Ks}\;$networks, quantified by neuron order within a burst, is compared runs on the same network realization have more similar burst structure than those on different realizations (Figure 2B, right).

**Figure 3:**
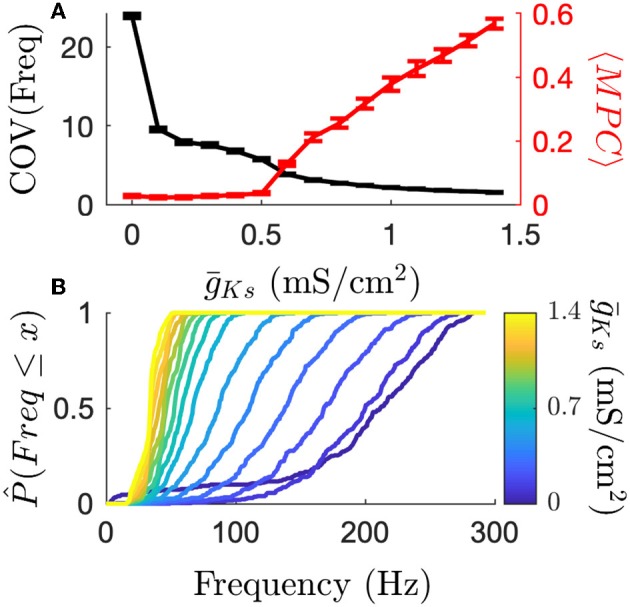
The transition from high frequency variance to high phase locking shows how cholinergic modulation can change coding principles. **(A)** High ACh networks have highly varied firing rates as measured by the coefficient of variation. Firing rates quickly become more uniform as ${\bar{g}}_{Ks}$ increases. Conversely, MPC (phase locking) is high for low ACh networks. **(B)** Frequency CDFs for single simulations, each on the same network structure, show that the same network display large differences in the variance of firing rates across the network. High ACh networks have high variance, which deceases dramatically as ACh is reduced. Error bars indicate s.e.m.

We next proceeded to quantify more carefully the underlying mechanisms of this coding switch. We first investigated the modulation of frequency variance and phase locking (as measured by mean phase coherence, MPC) with varying ACh as it provides a basis for the different coding schemes (Figure 3). We observed that high ACh networks have high frequency variance and low phase locking. As ACh is reduced (${\bar{g}}_{Ks}$ is increased), frequencies become more uniform, and phase locking increases. Figure 3 (top, black curve) shows that when ACh is high (${\bar{g}}_{Ks}$ is low) the firing rate distribution is wide as measured by coefficient of variation. As ACh is reduced firing rate variance rapidly decreases and all neuron firing rates collapse to the mean, which can be seen by comparing empirical cumulative distribution functions of firing rate on the same network for varying ${\bar{g}}_{Ks}$ (Figure 3, bottom). At the same time, the transition to phase locked firing happens for networks with low ACh (high ${\bar{g}}_{Ks}$) (Figure 3, top, red curve). This transition supports a transition between rate and phase coding regimes. These two effects on the character of network dynamics provide a substrate for each coding scheme at each pole of cholinergic modulation. High ACh networks are primed for rate coding and low ACh networks are primed for phase coding.

**Figure 4:**
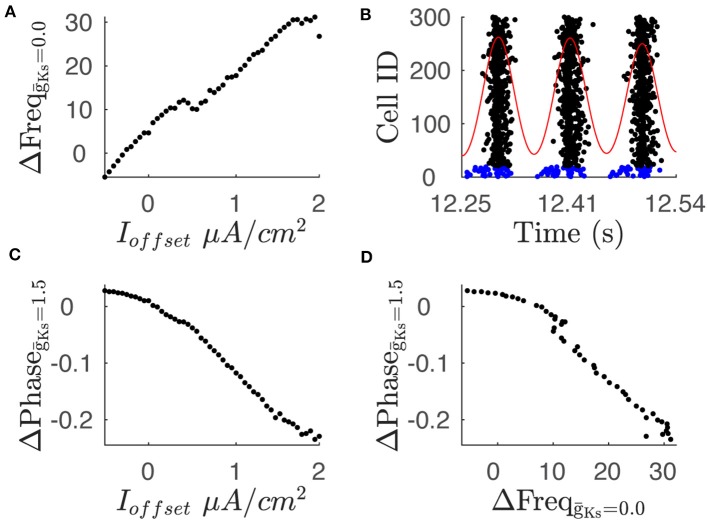
Variations in current input between neuron subsets leads to changes in average frequency and phase. **(A)** The difference in average frequency of the two neuron populations shows a positive relationship with the difference in current input, labeled as I_offset_, when ${\bar{g}}_{Ks}$ is 0.0. **(B)** Raster plot shows phase leading in spike times of neuron subset. The raster plot shows spike times for neuron population where 20 neurons receive an additional current input of 1.95 μA/cm^2^. Blue rasters indicate subpopulation with additional current while black rasters indicate sub population with baseline current input. Red trace shows convolution of spike times with Gaussian function which is used to define the phase reference. The above simulation is conducted with a ${\bar{g}}_{Ks}$ value of 1.5. **(C)** Phase difference between subpopulation with additional current input and subpopulation with baseline current input shows a negative relationship with the current input. **(D)** Comparison of the phase difference and frequency difference for a given current input. Plot shows comparison of phase difference for ${\bar{g}}_{Ks}$ = 1.5 and frequency difference for ${\bar{g}}_{Ks\;}$ = 0.0 for a given current offset.

We then investigated the effects of differential input on the network dynamics. Specifically, we measured the effect on network dynamics, in both regimes (high and low ACh), when randomly chosen neurons receive additional input current. We compared dynamics of networks with constant structure, where 20 excitatory neurons received an additional offset current I_offset_ (up to 1.95 μ*A*/*cm*^2^) above the remaining neurons. In these simulations, when ${\bar{g}}_{Ks}=0.0$ mS/cm^2^, increasing I_offset_ reliably increased the firing rate of the subset of neurons as expected (Figure 4A). When ${\bar{g}}_{Ks}=1.5$ mS/cm^2^, the subset of neurons fired at an increasingly earlier phase as I_offset_ increased (Figures 4B,C). The increase in firing during high ACh conditions and the phase advancement in the low ACh conditions are correlated (Figure 4D) providing a link between the two representations. The effect of differential input on frequency and phase form the basis for frequency coding when ACh is high and phase coding when ACh is low.

To further pursue our hypothesis and measure the extent that networks either rate code or phase code information about their structure, we investigated if the correlation in frequency (for Type 1, high ACh, low ${\bar{g}}_{Ks}$ conditions) or the phase locking (for Type 2, low ACh, high ${\bar{g}}_{Ks}$ conditions) between pairs of neurons would be more similar on repeated simulations of the same structural realization of randomly generated networks vs. different structural realizations. Similarly, for the dynamical response to external stimulation patterns in networks with fixed connectivity, we investigated if the correlation in frequency (under Type 1, high ACh, low ${\bar{g}}_{Ks}$ conditions) or the phase locking (for Type 2, low ACh, high ${\bar{g}}_{Ks}$ conditions) between pairs of neurons would be more similar on repeated simulations with the same DC input pattern to the network vs. different random realizations of DC input.

To measure these functional relationships between neurons, we constructed a similarity score based on three measures: pairwise mean phase, pairwise MPC, and frequency. As indicated before, MPC is a measure of phase locking between pairs of neurons and ranges between 0 for random firing and 1 for perfect phase locking. To compare two simulations, we define the phase similarity (S_Phase_) as the correlation in pairwise mean phase calculated across all neurons that fired 30 or more spikes in both simulations. Similarly, to compare neuron frequencies across simulations, the frequency similarity (S_Freq_) is defined as the correlation of their frequencies across both simulations. To maximize the variance within the data, principal component analysis was performed on the data for each level of ${\bar{g}}_{Ks}$, and the data was projected onto the 1st principal component for only correlation analysis. Calculation of MPC and coefficient of variation were performed on raw data.

Finally, the dependence of network firing pattern on network structure, or the pattern of DC inputs, was quantified by a network similarity score based on either frequency or phase, NS_Freq_ or NS_Phase_. This score was defined by:

$$\begin{array}{l} NS_{Freq,i}=\frac{<S_{Freq,i}>-<S_{Freq,\sim i}\;>}{2}, \end{array}$$

or:

$$\begin{array}{l} NS_{Phase,i}=\frac{<S_{Phase,i}>-<S_{Phase,\sim i}\;>}{2}, \end{array}$$

where *S*_*x,i*_ (*x* = *Freq* or *Phase*) is the similarity between all runs on the same realization of network structure *i* (or of DC input pattern *i*); *S*_*x*,~*i*_ is the similarity between the runs on network *i* and all other network realizations (or with DC input pattern *i* and all other DC input patterns). NS_*x*_ will be 1 if all runs on the same network (or input pattern) realization have identical network similarity while all other network (or input pattern) realizations are orthogonal, and it will be 0 if all runs are equally similar regardless of network structure (or DC input pattern). NS_*x*_ will be −1 if all runs on the same network (or DC input pattern) realization have orthogonal network similarity but it is identical on runs with different realizations.

To account for the effect of an increased bandwidth which results from a wider distribution of frequencies (i.e., a frequency pattern with a wider range will be easier to detect than a narrow pattern) NS_Freq_ was scaled by the coefficient of variation as such:

$$\begin{array}{l} {\tilde{NS}}_{Freq,i}=\frac{<COV_{Freq,i}>\left(<S_{Freq,i}>-<S_{Freq,\sim i}>\;\right)}{2}. \end{array}$$

Similarly, to account for low MPC reflecting random firing between neurons NS_Phase_ was scaled by the average MPC of each network across all simulations:

$$\begin{array}{l} {\tilde{NS}}_{Phase,i}=\frac{\langle MPC_{i}\rangle \left(<S_{Phase,i}>-<S_{Phase,\sim i}>\right)}{2} \end{array}$$

For simplicity, pairwise phase relationships between all excitatory neurons and an arbitrary inhibitory neuron were analyzed.

**Figure 5:**
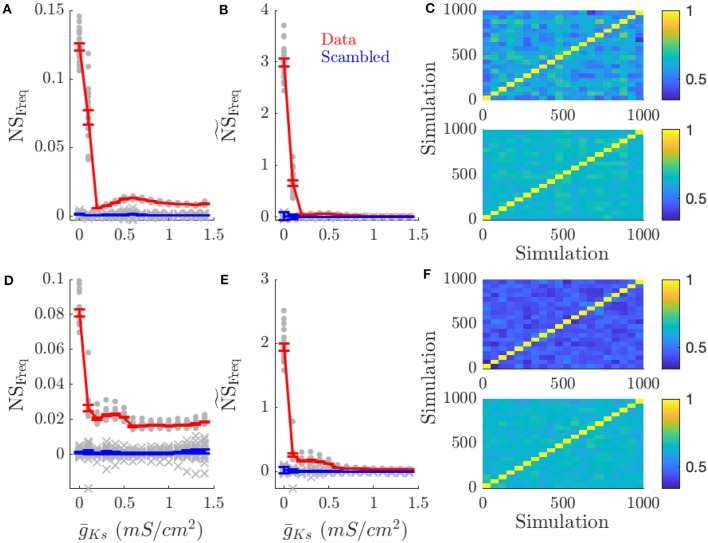
High ACh networks show increased rate coding which is diminished in low ACh networks. Rate coding, measured by the specificity of neuronal firing rates across simulations with the same pattern of inputs across the network vs. different patterns of input, occurs for high ACh networks. This effect is decreased in low ACh networks, largely because firing rates become more similar between different networks. **(A)** NS_Freq_ is the network score based on comparing frequency correlations on simulations with the same input pattern against simulations with different patterns. **(B)** ${\tilde{NS}}_{Freq}$ scales NS_Freq_ by the coefficient of variation for frequency. **(C)** Color plots show the correlation of firing rates between simulations for ${\bar{g}}_{Ks}=0.0$ and ${\bar{g}}_{Ks}=1.4$ mS/cm^2^ (top and bottom, respectively). Each simulation is sorted along the x and y axis by network structure. A similar effect occurs when information is represented through network structure. **(D–F)** NS_Freq_, ${\tilde{NS}}_{Freq}$, and correlation plots for simulations with varying network structure. Gray points show the NS_Freq_ for each input pattern or network structure. Gray crosses show NS_Freq_ for scrambled data. Error bars indicate s.e.m.

**Figure 6:**
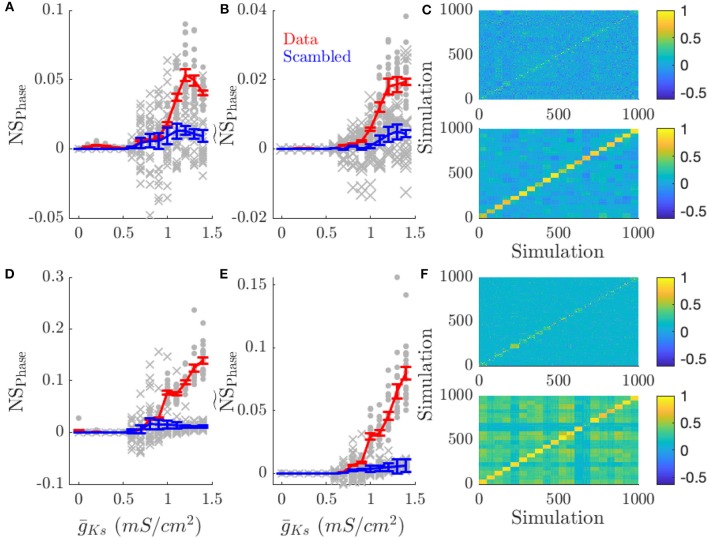
Low ACh networks show increased phase coding. Phase coding, measured by the network specificity of mean phase coherence across simulations with the same input pattern vs. different patterns, occurs for low ACh networks on all topologies. This effect is decreased in high ACh networks, due to the increased frequency variation and decreased phase locking. **(A)** NS_Phase_ is the network score based on phase correlations. **(B)** ${\tilde{NS}}_{Phase}$ scales NS_Phase_ by the mean MPC of the simulations. Scaling average MPC accounts for low MPC reflecting essentially random firing. **(C)** Color plots show the correlation of phase values between simulations for ${\bar{g}}_{Ks}=0.0$ and ${\bar{g}}_{Ks}=1.4$ mS/cm^2^ (top and bottom, respectively). Each simulation is sorted along the x and y axis by network structure. **(D–F)** NS_Phase_, ${\tilde{NS}}_{Phase}$, correlation plots for simulations with varying network structure. Gray points show the NS_Phase_ for each input pattern or network structure. Gray crosses show NS_Phase_ for scrambled data. Error bars indicate s.e.m.

We measured mean frequency similarity score, NS_Freq_, for both cases, networks with changing DC input patterns (Figures 5A–C) and changing connectivity structure (Figures 5D–F), and we measured phase similarity score NS_phase_, for the two cases (Figures 6A–C and Figures 6D–F, respectively). The results in both figures are compared to scrambled spike trains (blue line).

Rate coding of DC input pattern (Figures 5A–C) and network structure (Figures 5D–F) was prevalent for high ACh (low ${\bar{g}}_{Ks}$) dynamics and reduced for low ACh (high ${\bar{g}}_{Ks}$) dynamics. As ${\bar{g}}_{Ks}$ increased (lower ACh), network frequency similarity scores decreased (first and middle columns), but not because of reduced frequency correlations within the same input pattern (last column). Instead neuronal frequencies across other network realizations became more correlated. This is evident in the frequency correlations for runs on the same realization of an input pattern or network structure compared to runs with other patterns or structures (diagonal vs. off-diagonal elements in Figures 5C,F, respectively). This is expected as all frequencies across all network realizations converge due to reduced gain of the f/I curve for Type 2 cells.

In low ACh (high ${\bar{g}}_{Ks}$) conditions, networks have highly phase locked dynamics and phase coding prevails (Figure 6). Networks presented with the same pattern of inputs (Figures 6A–C) or having the same network structure (Figures 6D–F) showed higher network phase similarity scores as ${\bar{g}}_{Ks}$ increased (first and middle columns), and displayed higher phase correlations than between different patterns or structures when ${\bar{g}}_{Ks}$ is high (low ACh) (color plots in Figures 6C,F). As ACh is reduced (and ${\bar{g}}_{Ks}$ is correspondingly increased) correlations in firing phase decrease. This effect is apparent in NS_Phase_ (Figures 6A,D) and becomes even more pronounced when phase-locking is taken into account (Figures 6B,E). As opposed to frequency correlations, phase correlations are uniformly low for simulations where different patterns were presented (Figure 6C). This effect is due to the low phase locking in high ACh conditions.

Finally, we checked robustness of the obtained results by introducing and varying the level of external noise to the networks and also, separately, by changing the strength of excitatory coupling, which effectively changes internal excitatory-inhibitory balance within the network. Rate coding during high ACh conditions and phase coding for low ACh was robust to changes in noise and variations in excitatory coupling. Figures S1, S2 show the effect of increasing noise on frequency coding (Figure S1) and phase coding (Figure S2). When information is presented as the pattern of DC input, in high ACh networks frequency coding is robust to increasing noise and was uniformly low for low ACh networks. Phase coding of inputs is more sensitive to noise at high ${\bar{g}}_{Ks}$, and uniformly low for low ${\bar{g}}_{Ks}$ (Figure S2).

As excitatory coupling was scanned from zero to 0.04 mS/cm^2^, frequency coding of inputs initially decreased as coupling was increased for all ${\bar{g}}_{Ks}$, but for low ${\bar{g}}_{Ks}$ networks frequency coding recovered (Figure S3). Low ${\bar{g}}_{Ks}$ networks maintained a higher NS_Freq_ as coupling increased compared to high ${\bar{g}}_{Ks}$ networks. When representing network structure, coupling needed to reach a sufficient level for frequency coding to occur. Phase coding, on the other hand, required a minimum coupling strength to emerge (Figure S4), but only emerged for high ${\bar{g}}_{Ks}$. For the highest ${\bar{g}}_{Ks}$ phase coding of both network structure and input pattern began to decrease.

## Discussion

Using the Ks model we have shown that neuromodulation of the M-current can switch networks from a rate coding regime when ACh is high (${\bar{g}}_{Ks}$ is low) to an oscillatory phase coding state when ACh is low (${\bar{g}}_{Ks}$ is high). This neuronal model recreates biophysical changes displayed in neurons when the muscarinic system is activated, including gain modulation, PRC modulation, and SFA modulation (Figure 1). As ACh levels are continuously changed, these three properties are inflected over different ranges of the maximal conductance of the I_M_, ${\bar{g}}_{Ks}$.

We note that here we focus only on the biophysical effects of a single target of ACh modulation, inactivation of slow (M-type) potassium channels. However, ACh has numerous effects both at the cellular and network level. Through the nicotinic receptors ACh directly depolarizes neurons and the nicotinic signal is faster than the cascade required to inactivate M-type potassium channels. Activation of muscarinic channels also inhibits presynaptic release at both excitatory and inhibitory axon terminals reducing the effects of recurrent connectivity (Hsieh et al., 2000; Hasselmo, 2006). During high ACh release direct depolarization of neurons through nicotinic receptors, reduction in local network inputs, increased gain, and reduced SFA could all work together to prime a network to represent an external input in the firing rate distribution. Removal of these effects would the lead to phasic dynamics shaped the structure of the local network.

It is important to note that all three of I_M_ modulated properties, gain modulation, PRC modulation, and SFA modulation, are important for switching from a rate to a phase coding regime. For rate coding in high ACh conditions, high gain is beneficial in widening the firing rate distribution for a given range of synaptic inputs. Low SFA allows neurons to persist in firing to maintain a representation in frequency space and low synchrony facilitated by Type 1 PRC, prevents a reduction in frequency variation. For phase coding under low ACh conditions, low gain reduces frequency variation in the network, while a Type 2 PRC and high SFA induce increased periodicity and synchronizability for phase differences to persist.

Thus, reductions in ACh level provide two dynamical substrates for phase coding: (1) near uniformity in firing rates across the network, and (2) the ability of neurons to collectively organize into network-wide oscillatory behavior.

By directly quantifying the dependence of a network firing pattern on a particular network realization for networks of the same connection structure and external input pattern we've provided strong evidence that Type 1 networks represent information about internal structure and external inputs through rate coding (Figure 5) while Type 2 networks' firing patterns provide oscillatory phase coding dynamics (Figure 6).

The fact that the transition from rate coding to phase coding firing patterns occurs over the ${\bar{g}}_{Ks}$ range when the gain of the neuron (f/I curve) is significantly modulated, points to the importance of this property for switching coding regimes. When a network of high gain neurons is formed, slight variations in synaptic input will result in higher firing rate differences between neurons. This wide, input dependent, firing rate distribution will drive the network firing rate distribution and be reproducible for a given set of inputs or a given network structure. As gain is reduced, frequency differences between neurons will be reduced allowing neuronal properties such as SFA and PRC effects to impact network dynamics in a significant way. For example, it is well-known that networks of periodic oscillators synchronize easier when the frequency range is reduced and that large variance in frequencies promotes the formation of discrete clusters of synchronization (Osipov and Suchchik, 1998; Acebrón et al., 2005; Favaretto et al., 2017).

Spike initiation dynamics and the adaptation mechanics of neurons have been suggested as being substrates for coding through integration or coincidence detection (Prescott and Sejnowski, 2008; Ratté et al., 2013). While both integrative and coincidence coding can exist with wide firing rate distributions, phase coding relies on neurons being close in frequency while high neuronal gain facilitates rate coding (Gjorgjieva et al., 2014). The importance of co-modulation of neuronal gain and excitability type in transitioning a network from rate to phase coding is an essential result of the present work.

Gain modulation improves signal recognition in a variety of brain regions (Atiani et al., 2009; Schäfer et al., 2009; Williamson et al., 2016; Angeloni and Geffen, 2018). In many cases gain modulation is attributed to fluctuations in synaptic inputs and synaptic plasticity due to gain modulation being stimulus dependent (Chance et al., 2002; Cardin et al., 2008; Carvalho and Buonomano, 2009). But changes in ACh tone also change the gain response of neurons (Barkai and Hasselmo, 1994; Soma et al., 2012, 2013). ACh release is increased when an animal is performing an attentional task and its release is correlated with task performance (Himmelheber et al., 2000; Kozak et al., 2007; Parikh et al., 2007). These results point to cholinergic modulation priming neuronal networks to respond with an appropriate rate code to a given cue by increasing the gain of the neurons. This also indicates that rate coding may be better at facilitating representations of sensory information than phase coding.

The Type 2 dynamics of the low ACh state support robust synchronized bursting required for oscillations in population activity (Bogaard et al., 2009; Fink et al., 2011; Knudstrup et al., 2016). ACh release is important for the generation of the theta rhythm in the hippocampus (Vertes and Kocsis, 1997; Hasselmo, 2006; Alger et al., 2014). But a temporal analysis of both ACh release and theta band power shows that peaks in ACh release lag behind increases in theta power (Zhang et al., 2010). This suggests that ACh release is actually working to disrupt synchrony within the theta oscillation. Further evidence for the role ACh release could play in reducing synchronous firing is seen in its suppression of sharp wave ripples (Hasselmo, 2006).

Changes in coding modality, in addition to affecting information transfer to downstream targets, would have a profound effect on learning through activity dependent synaptic plasticity. STDP has a strong frequency dependence, even with random spike trains (Sjöström et al., 2001; Wittenberg and Wang, 2006). When spike pairs are presented at a high frequency synapses have net potentiation, but have net depression for low frequency. When networks are in the high ACh rate coding regime, this would lead to highly activated neurons forming a strongly connected cluster within the networks, which would reinforce the specific frequency pattern imposed by an external stimulus. During the low ACh phase coding regime the stable phase relationships would shape synaptic plasticity. The reduction in mean frequency would lead to a net reduction in synaptic weight (Sjöström et al., 2001), and the synchronization and resonance properties of neurons in the low ACh state preferentially strengthen connections from neurons with high input to neurons with low input (Fink et al., 2013; Roach et al., 2018). A complicating factor in interpreting the effects of ACh on network coding is that ACh significantly modulates STDP itself, acting as a gate on the LTD component, thus reducing the plasticity effects during low ACh conditions (Seol et al., 2007).

ACh release is very closely related to the sleep-wake cycle. ACh release is highest during wakefulness and rapid eye movement (REM) sleep and lower during non-REM (NREM) sleep (Marrosu et al., 1995). When the Ks model simulates these levels of ACh it recreates similar changes in spiking dynamics that are seen across these states (Roach et al., 2015). Within the context of the effects of ACh on network dynamics, we hypothesize that the high ACh waking state highlights the variance in magnitudes of external inputs to the given circuit in terms of neuronal frequency responses and primes networks to encode these inputs as stable patterns in frequency space subsequently storing this representation within synaptic weights. Elevated firing frequency and representations in frequency space may be important for the rapid encoding of the memory backbone and for transfer of information to other brain regions. In NREM sleep, when no external input is present and firing frequency distributions across neurons homogenize (Miyawaki et al., 2019), oscillatory dynamics pairs with phase coding to represent stored information as spike time differences between neurons which could facilitate consolidation of stored memories from a small group of neurons with strong synaptic inputs to the network as a whole (Puentes-Mestril et al., 2019). Additionally, ACh effects on synaptic plasticity, namely high ACh leads to increases in average synaptic weights and low ACh decreases them, support the synaptic homeostasis hypothesis (Tononi and Cirelli, 2003; Fink et al., 2013), but at the same time the proposed shift in the coding schemes paints a more complex picture of specific roles of sleep cycles. The widening of neuronal firing rate distributions across sleep-wake states also indicates that gain modulation by ACh is shaping network activity (Mizuseki and Buzsáki, 2013).

The role of ACh level in sleep dependent memory consolidation and synaptic homeostasis suggests that changes in coding modality may be optimized for storage of information in various encoding/behavioral states. Namely, during waking, high ACh conditions lead to enhancing the connections between neurons which receive the most input, forming a tightly connected cluster which forms the kernel of a new memory. In the sleep that follows, cycles of phase coding NREM distributing this kernel throughout the network are paired with cycles of REM reinforcing the distributed memory by re-enhancing connections to the neurons most active during REM bouts. Thus, ACh modulating the coding regime across behavioral states may facilitate an iterative process by the sleep cycle to tune memory consolidation (Puentes-Mestril et al., 2019).

Thus, we propose that ACh is a neuromodulator that is critical for memory consolidation throughout the brain. The biophysical changes in neural excitability that the I_M_ governs lead to significant changes in the spiking and oscillatory processes in the brain. The effects of gain modulation in switching between circuit activity that has high or low dependence on network structure or external input pattern may be central to ACh's role in information processing at the network level. Additionally, the dynamic nature of ACh release could allow for a stable network to coordinate information processing functions across various brain states. While ACh has other pathways of neuromodulation, notably through the nicotinic receptor which directly depolarizes neurons (Sarter et al., 2014), we show that the muscarinic effects of changing ACh levels are sufficient to change coding modes.

## Methods

Networks were composed 300 excitatory and 75 inhibitory neurons arrayed on two interconnected ring lattices. Excitatory neurons were randomly connected to 3% of the neurons on each lattice, while inhibitory neurons were connected to 6%. The random process used in the generation of a network structure was seeded such that one of 20 network structures could be reproduced.

Connections between neurons were defined by a synaptic conductance pulse:

$$\begin{array}{lll} g_{syn,ij}\left(t\right) & = & \text{max}({\left(e^{\frac{-\left({\tilde{t}}_{\max}-\tau_{D}\right)}{\tau_{S}}}-e^{\frac{-\left({\tilde{t}}_{\max}-\tau_{D}\right)}{\tau_{F}}}\right)}^{-1}\\ \; & \; & \left(e^{\frac{-\left({\tilde{t}}_{j}-\tau_{D}\right)}{\tau_{S}}}-e^{\frac{-\left({\tilde{t}}_{j}-\tau_{D}\right)}{\tau_{F}}}\right),0)\;\\ {\tilde{t}}_{\max} & = & \frac{\left(\tau_{D}\tau_{S}-\tau_{D}\tau_{F}-\tau_{S}\tau_{F}\ln\left(\frac{\tau_{F}}{\tau_{S}}\right)\right)}{\left(\tau_{S}-\tau_{F}\right)} \end{array}$$

where ${\tilde{t}}_{j}$ is the time of the last spike fired by the presynaptic neuron *j*, ${\tilde{t}}_{\max}$ is the time where the synaptic pulse reaches its maximum, τ_*D*_ is a synaptic delay constant set to 0.08 ms, τ_*S*_ is the slow synaptic decay constant set to 3 ms, and τ_*F*_ is the fast synaptic decay constant set to 0.3 ms. Thus, the synaptic pulse ranges between 0 and 1. The total synaptic input to a neuron *i* is defined by:

$$\begin{array}{lll} I_{syn,i}\left(t\right) & = & w_{e}\overset{N_{E}}{\underset{j=1}{\sum }}A_{ij}g_{syn,ij}\left(t\right)\left(E_{E}-V_{i}\right)\\ \; & + & w_{i}\overset{N_{I}}{\underset{j=1}{\sum }}A_{ij}g_{syn,ij}\left(t\right)\left(E_{I}-V_{i}\right) \end{array}$$

where *V*_*i*_ is the membrane potential of neuron *i*, *E*_*E*/*I*_ is the reversal potential of either excitatory or inhibitory synapses, *A*_*ij*_ is 1 if neuron *j* synapses onto neuron *i* or 0 otherwise, and *N*_*E*/*I*_ is the number of excitatory or inhibitory neurons. The synaptic weight, *w*_*e*/*i*_, was set to 0.02 mS/cm^2^ for all simulations, unless otherwise stated.

In the Ks neuron model, the membrane potential evolved according to:

$$\begin{array}{lll} c_{m}\frac{dV_{i}}{dt} & = & I_{syn,i}+I_{ext,i}-m_{\infty }^{3}h{\bar{g}}_{Na}\left(V_{i}-E_{Na}\right)-n^{4}{\bar{g}}_{Kdir}\left(V_{i}-E_{K}\right)\\ \; & - & s{\bar{g}}_{Ks}\left(V_{i}-E_{K}\right)-g_{L}\left(V_{i}-E_{L}\right), \end{array}$$

where ${\bar{g}}_{x}$ is the maximal conductance associated with an ionic current, *E*_*x*_ is the reversal potential for an ion, and *I*_*ext, i*_ is a random direct current which is unique to each neuron and constant during a simulation. The range of *I*_*ext, i*_ was set to 2.0 μA/cm^2^ and the mean was set so that neurons would fire at 10 Hz in the absence of any other inputs. The uniform random process which generated patterns of *I*_*ext, i*_ was seed so that one of 20 unique patterns could be reproduced.

The gating variables *h, n*, and *s* were of the form

$$\begin{array}{l} \frac{dx}{dt}=\frac{\left(x_{\infty }\left(V\right)-x\right)}{\tau_{x}\left(V\right)}, \end{array}$$

where *x*_∞_(*V*) is the steady state value of the variable and τ_*x*_(*V*) is the time constant. τ_*s*_(*V*) = 75 ms for all *V*. When *V*_*i*_ crossed 0 mV a spike was recorded and synaptic outputs were triggered. ${\bar{g}}_{Ks}$ is the parameter responsible for the transitions in excitability seen in this model and is used as a proxy for the level of acetylcholine (which is inversely proportional to ${\bar{g}}_{Ks}$).

Noise was introduced by randomly inducing spiking in all neurons at a low rate. Unless otherwise noted the frequency of noise was set to 1 Hz. All simulations were integrated for 7 s at a 0.05 ms time step using a 4th order Runge-Kutta algorithm. This neuronal dynamic was taken from Stiefel et al. (2008b), for more details see Fink et al. (2011).

Spiking dynamics from 20 patterns of external current input or 20 network structures were analyzed. Each pattern or network was simulated 50 times. The network activity was quantified in two ways. The first was to calculate an average firing rate for neurons in a network. To maximize the variance in the data principal component analysis was performed on the frequencies for all simulations run at a given ${\bar{g}}_{Ks}$ and the frequencies were projected onto the 1st principal component for further analysis. The correlation of firing rates (in PCA space) between simulations was calculated as the dot product of a vector containing frequencies sorted by cell id for each simulation, yielding the value *S*_*F*_. All analysis considered only spikes which fired during the last 5 s of the simulation. Phase correlations were calculated in a similar manner. As a control, the frequencies/phases were scrambled and assigned to random neurons.

Whether, a network was in a phase coding regime was based on a measure of how stable the phase relationships are between neurons in the network. For a pair of neurons *i* and *j* the phase relationship for a given (the *kth*) spike fired by neuron *i* is $\phi_{ij}^{k}=2\pi \frac{\left(t_{k}^{i}-t_{-}^{j}\right)}{\left(t_{+}^{j}-t_{-}^{j}\right)}$, where $t_{k}^{i}$ is the time of the *k*^*th*^ spike fired by cell *i*, $t_{-}^{j}$ is the time of the last spike fired by neuron *j* before $t_{k}^{i}$, and $t_{+}^{j}$ is the time of the first spike fired by neuron *j* after $t_{k}^{i}$. Between neurons *i* and j the phase coherence, or how reliable the phase difference between the neurons is across cycles, is:

$$\begin{array}{l} r_{ij}=\frac{1}{T}\sum_{k=\;0}^{T}e^{i\phi_{ij}^{k}}, \end{array}$$

where *T* is the number of spikes fired by neuron *i* that are between a pair of spikes fired by neuron *j*. Note that this measure is not reciprocal (i.e., *r*_*ij*_ = *r*_*ji*_ is not always true). The pairwise *r*_*ij*_ values are averaged for all neurons which fire more than 30 spikes in the simulation and for presentation the average value of each network was computed.

Network bursts were identified by binning spike times into 0.05 ms time bins and convolving with a Gaussian kernel $k\left(t\right)=e^{\frac{{-t}^{2}}{\sigma^{2}}}$ evaluated between −10 and 10 ms and with σ^2^ of 1 ms. When the convoluted signal was above a threshold of 10% of the neurons in the network the network was considered to be in a burst. Each burst was padded with 10 ms before and after the threshold crossing to any capture the spikes initiating the burst. To evaluate the similarity of two bursts the order of neuron's firing in a burst window was correlated. Example bursts were selected by identifying those bursts with the highest correlation on two different runs on the same network realization. Average pairwise burst correlations were compared between two runs on the same network realization and one run on two different realizations.

## Data Availability Statement

The simulation code used to generate data for this study can be found on GitHub (https://github.com/Zochowski-UMnerualnetworks-lab/Rate_vs_phase_coding).

## Author Contributions

JR, BE, VB, LS, and MZ designed research. JR and BE performed research and analyzed data. JR, VB, LS, and MZ wrote the paper.

### Conflict of Interest

The authors declare that the research was conducted in the absence of any commercial or financial relationships that could be construed as a potential conflict of interest.
