# Supplementary material for: Acetylcholine Mediates Dynamic Switching Between Information Coding Schemes in Neuronal Networks
Source: Front Syst Neurosci. 2019 Nov 12;13:64. doi: 10.3389/fnsys.2019.00064 (PMC6861375; doi:10.3389/fnsys.2019.00064)
Supplement: Supplementary file 1 [file Table_1.DOCX]

Supplementary Material

# Supplementary Figures and Tables

## Supplementary Figures


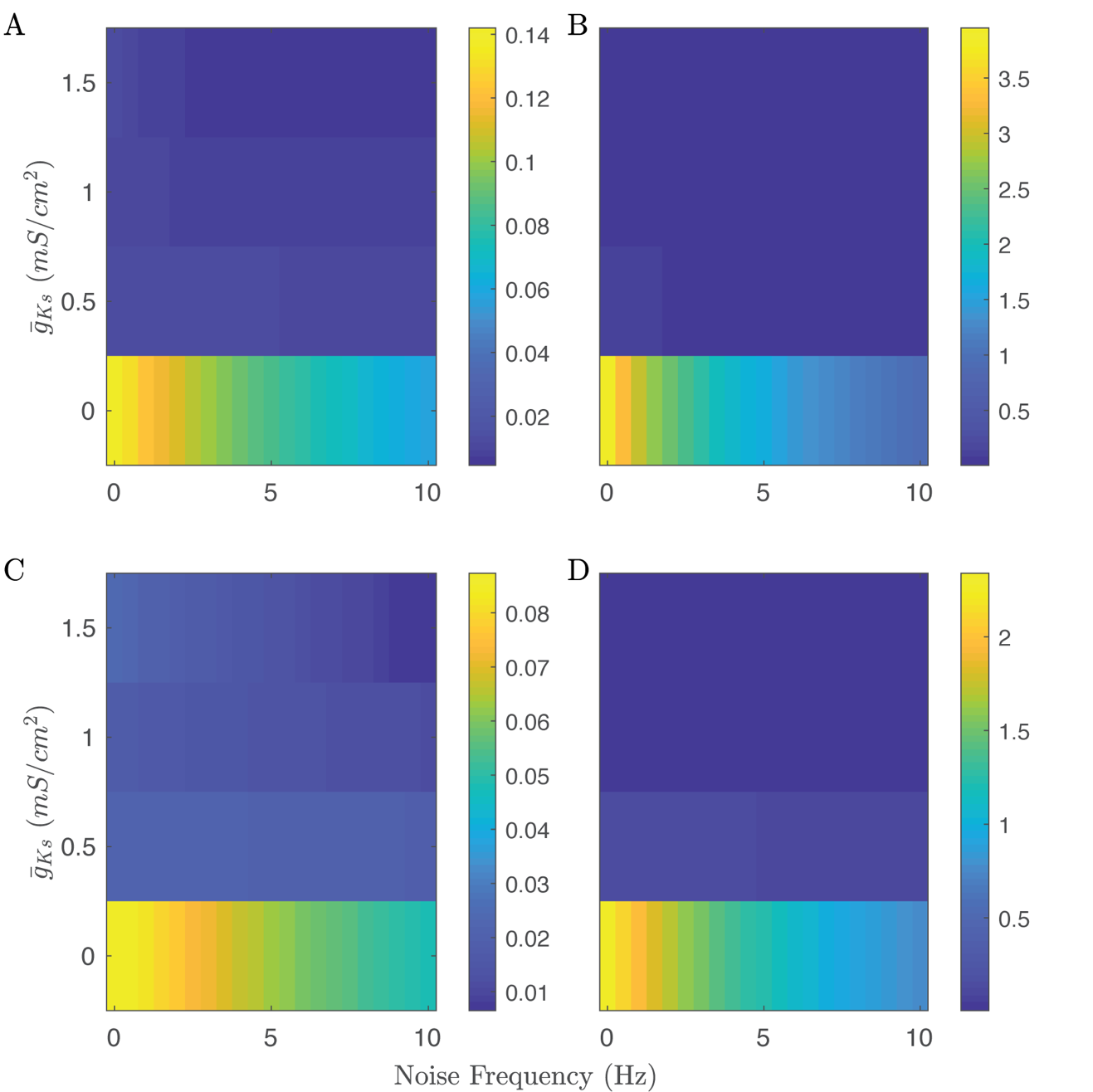


**Supplementary Figure 1.** Network frequency similarity scores for increasing noise with external input patterns (A&B) and on different networks (C&D). Low $\bar{g}_{Ks}$ networks rate code better than high $\bar{g}_{Ks}$ networks over all noise levels (A) NS_Freq_ and (B) $\tilde{NS}_{Freq}$ are shown for input patterns. (C) NS_Freq_ and (D) $\tilde{NS}_{Freq}$ for varying network structure.

**
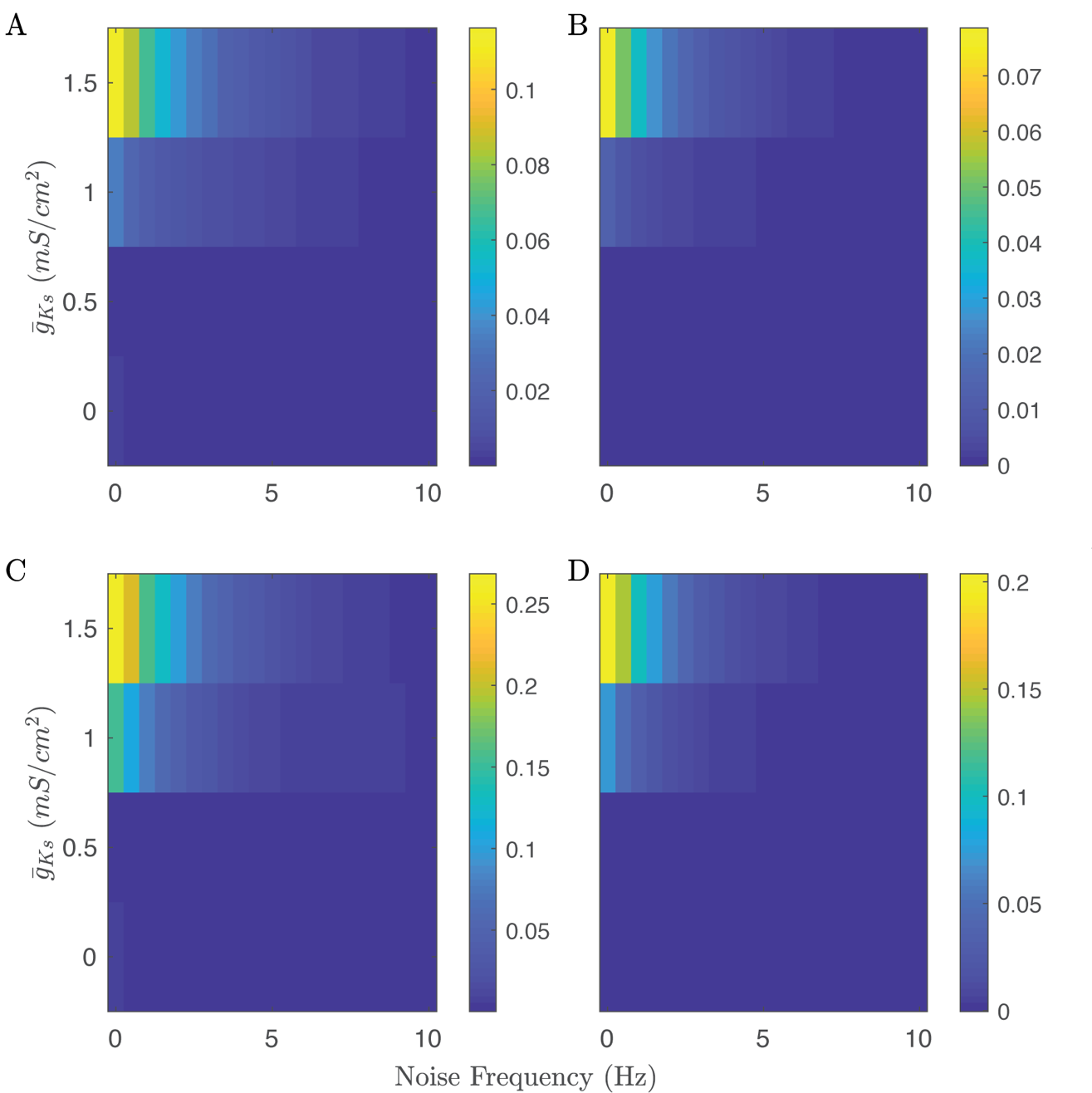
**

**Supplementary Figure 2.** Network phase similarity scores for increasing noise for external input patterns (A&B) and different network structures (C&D). High $\bar{g}_{Ks}$ networks phase code better than low $\bar{g}_{Ks}$ networks, but phase coding overall is highly noise dependent. (A) NS_Phase_ and (B) $\tilde{NS}_{Phase}$ for input pattens. (A) NS_Phase_ and (B) $\tilde{NS}_{Phase}$ for for network structures.

**
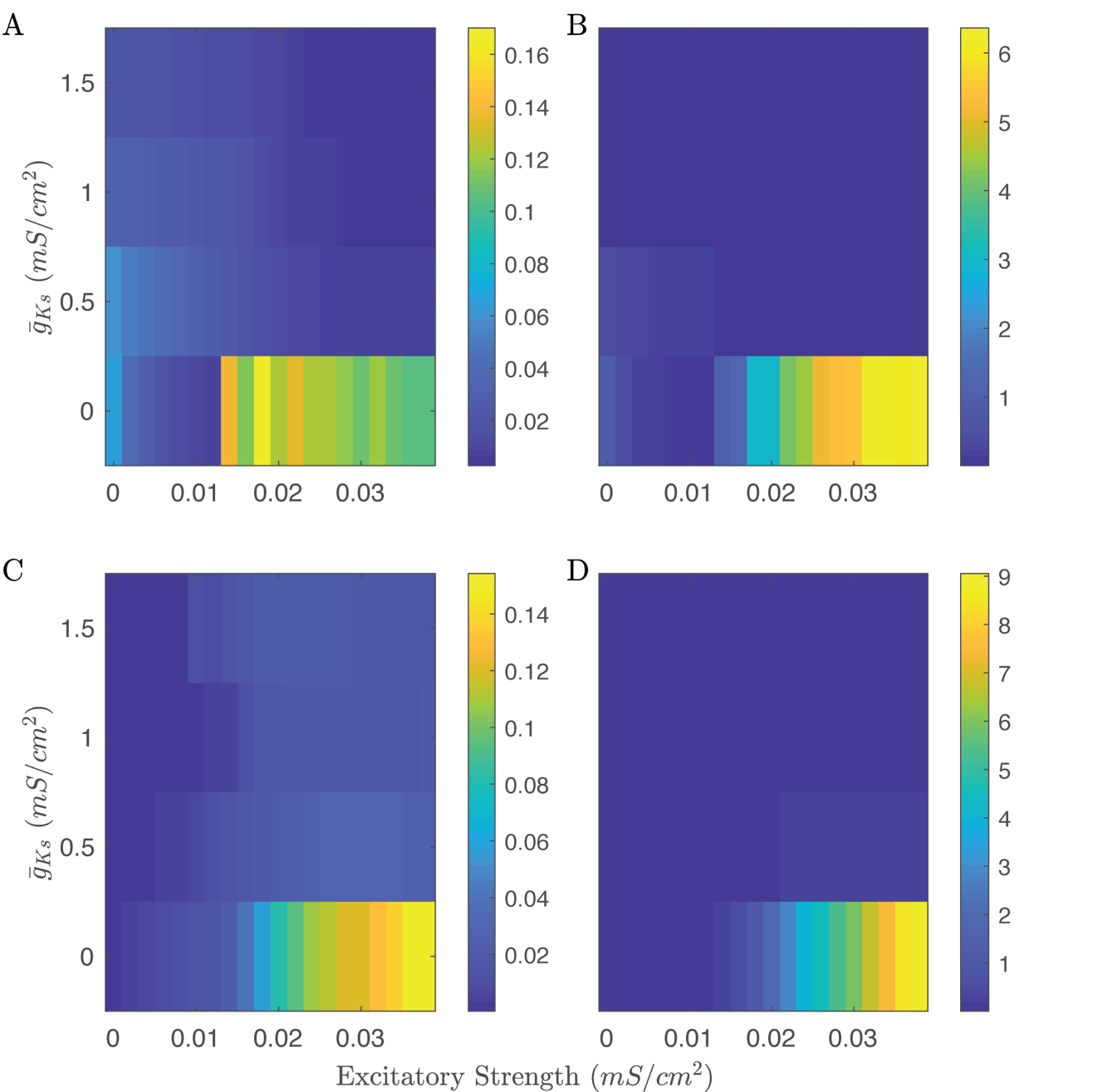
**

**Supplementary Figure 3.** Network frequency similarity scores for increasing excitatory coupling with external inputs (A&B) and network structures (C&D). Low $\bar{g}_{Ks}$ networks rate code better than high $\bar{g}_{Ks}$ networks with increasing performance as coupling increases. (A) NS_Freq_ and (B) $\tilde{NS}_{Freq}$ for input patterns. (A) NS_Freq_ and (B) $\tilde{NS}_{Freq}$ for network structures.

**
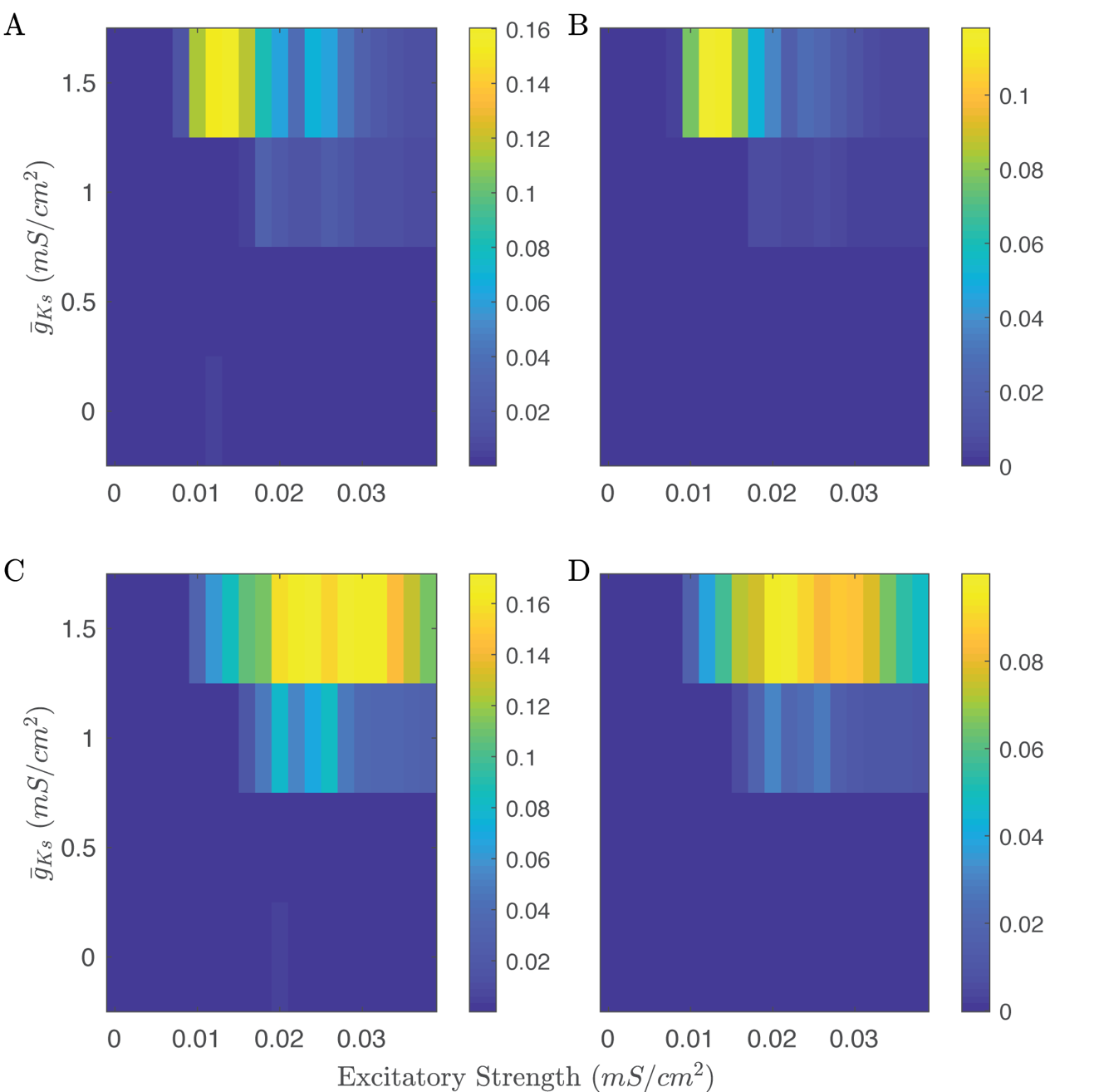
**

**Supplementary Figure 4.** Network phase similarity scores for increasing excitatory coupling with varying inputs (A&B) and network structures (C&D). High $\bar{g}_{Ks}$ networks phase code better than high $\bar{g}_{Ks}$ networks once coupling reaches a sufficient level and decreases for vary strong coupling. (A) NS_Phase_ and (B) $\tilde{NS}_{Phase}$ for input patterns. (A) NS_Phase_ and (B) $\tilde{NS}_{Phase}$ for network structures.
